# Ag Intercalation in Layered Cs_3_Bi_2_Br_9_ Perovskite for Enhanced Light Emission with Bound Interlayer Excitons

**DOI:** 10.1021/jacs.4c03191

**Published:** 2024-07-10

**Authors:** Anupam Biswas, Andrew J. E. Rowberg, Pushpender Yadav, Kyeongdeuk Moon, Gary J. Blanchard, Kyoung E. Kweon, Seokhyoung Kim

**Affiliations:** † Department of Chemistry, Michigan State University, East Lansing, Michigan 48824, United States; ‡ Quantum Simulations Group and Laboratory for Energy Applications for the Future (LEAF), Lawrence Livermore National Laboratory, Livermore, California 94550, United States

## Abstract

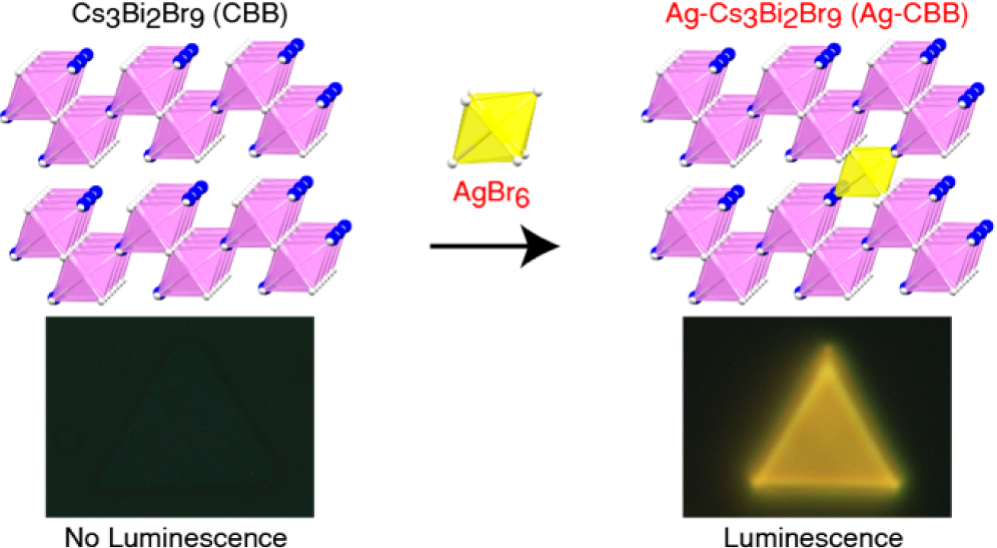

Cesium bismuth bromide (CBB) has garnered considerable attention as a vacancy-ordered layered perovskite with notable optoelectronic applications. However, its use as a light source has been limited due to its weak photoluminescence (PL). Here, we demonstrate metal intercalation as a novel approach to engineer the room-temperature PL of CBB using experimental and computational methods. Ag, when introduced into CBB, occupies vacant sites in the spacer region, forming octahedral coordination with surrounding Br anions. First-principles density functional theory calculations reveal that intercalated Ag represents the most energetically stable Ag species compared to other potential forms, such as Ag substituting Bi. The intercalated Ag forms a strong polaronic trap state close to the conduction band minimum and quickly captures photoexcited electrons with holes remaining in CBB layers, leading to the formation of a bound interlayer exciton, or BIE. The radiative recombination of this BIE exhibits bright room-temperature PL at 600 nm and a decay time of 38.6 ns, 35 times greater than that of free excitons, originating from the spatial separation of photocarriers by half a unit cell separation distance. The BIE as a new form of interlayer exciton is expected to inspire new research directions for vacancy-ordered perovskites.

## Introduction

Lead halide perovskites (LHPs) have been extensively investigated over the last few decades owing to their outstanding optoelectronic properties, chemical and structural diversity, and their ability to host functional impurities.^1,2^ Reducing the crystal dimensions from 3D networks to 2D quantum sheets^3^ enhances the radiative recombination of charge carriers due to reduced charge screening and an increased exciton binding energy,^4−6^ thereby demonstrating great promise for future light-emitting applications.^7^

Cesium bismuth bromide (CBB) was historically known as cesium enneabromodibismuthate^8,9^ (Cs_3_Bi_2_Br_9_) and has recently drawn considerable attention among the class of vacancy-ordered all-inorganic layered perovskites.^10−12^ However, despite the 2D structure, the photoluminescence (PL) of CBB has been reported to be extremely weak or near-dark at room temperature (RT),^10,13−15^ due to large electron–phonon coupling and a low-lying indirect transition slightly below the direct bandgap.^13^ While emission from a self-trapped exciton (STE) state formed by an off-centering distortion of [BiBr_6_]^3−^ octahedra is present, it is predominantly observed at temperatures below 60 K, where highly distorted [BiBr_6_]^3−^ octahedra become immobile.^10,16^ The cryogenic conditions needed to enable light emission significantly limit the use of CBB in practical light-emitting applications.

Various strategies have been employed to engineer luminescent properties of halide perovskites.^17^ Introducing dopants such as transition metals and lanthanides can provide additional emission bands, which involves an energy/charge transfer mechanism from the band edges of the hosts.^18−20^ Alloying is also widely used to enhance luminescence by either modifying the parity condition of radiative transitions by breaking local crystal symmetries^21,22^ or producing new STE bands by polaronic charge localization.^23^ For lighting applications, these approaches are often employed together to maximize the luminescence enhancement.^24,25^ While brightening of an intrinsic STE emission of CBB has been reported by the introduction of substitutional dopants,^16^ we have not found many successful cases of emission engineering of CBB.

In this study, we demonstrate a substantial enhancement of RT PL in single-crystalline CBB microcrystals through intercalation of Ag into the interlayer spacer region using chemical vapor deposition (CVD). CVD^26^ has a strong track record of achieving materials growth with unusual and often nonequilibrium characteristics in crystal structure,^27^ dopant incorporation,^28^ and morphologies,^6^ thereby unlocking new possibilities in materials research. Our CVD-grown Ag-intercalated CBB (Ag-CBB) exhibits bright yellow PL at ∼600 nm while retaining the crystal structure of the CBB host lattice. Using density functional theory (DFT) calculations, we find that Ag preferentially incorporates in the CBB lattice as an intercalant in the interlayer spacer regions, rather than as a substitutional impurity on nominal Cs or Bi lattice sites. The intercalated Ag forms an octahedral coordination with surrounding Br atoms, distorting the Bi–Br bonds of CBB, and exhibits a strong electron-trapping character. Upon photoexcitation, Ag quickly traps a photoelectron to form a small polaron within the spacer layer. The electron polaronically localized on Ag then forms an exciton with a hole remaining in the CBB layers, which we describe as a *dopant-bound interlayer exciton*. Radiative recombination of the bound interlayer excitons (BIEs) is responsible for the bright PL with a large Stokes shift of 0.75 eV from the CBB bandgap of 2.62 eV. In addition, Ag-CBB exhibits a prolonged radiation decay time of ∼38.6 ns, which is ∼35 times longer than the free exciton decay time of ∼1.1 ns. The significant increase in lifetime results from the spatial separation of charge carriers between the intercalated Ag and the host layer.

## Results

### Structural Analysis

**Figure 1:**
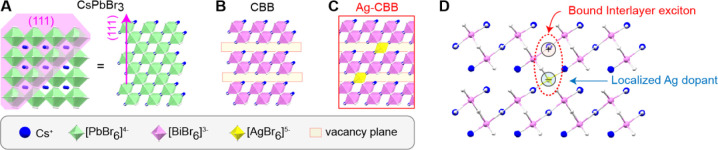
Crystal structure of Ag-CBB and bound interlayer exciton. (A−C) Crystal structures of prototypical cubic CsPbBr_3_ (A), vacancy-ordered CBB (B), and Ag-CBB (C). (D) Schematic depiction of a bound interlayer exciton, an electron trapped on Ag coupled with a hole in the neighboring layer.

The crystal structures of CBB and Ag-CBB are schematically presented in Figure 1. We begin our discussion with CsPbBr_3_, a prototypical cubic, all-inorganic LHP, and view it from a sideways perspective to show stacking along the (111) direction. When divalent Pb^2+^ is replaced by trivalent Bi^3+^ to form CBB, the mismatching oxidation states are compensated by ordered layers of vacancies, or vacancy planes, formed in every two other (111) planes of the cubic lattice. As a result, CBB exhibits a layered crystal structure composed of bilayers of corner-sharing [BiBr_6_]^3−^ octahedra, as shown in Figure 1B. Ag, when introduced into the CBB lattice, is expected to occupy these vacancy sites in the spacer layer readily and to establish an octahedral coordination with surrounding Br atoms (Figure 1C).

**Figure 2:**
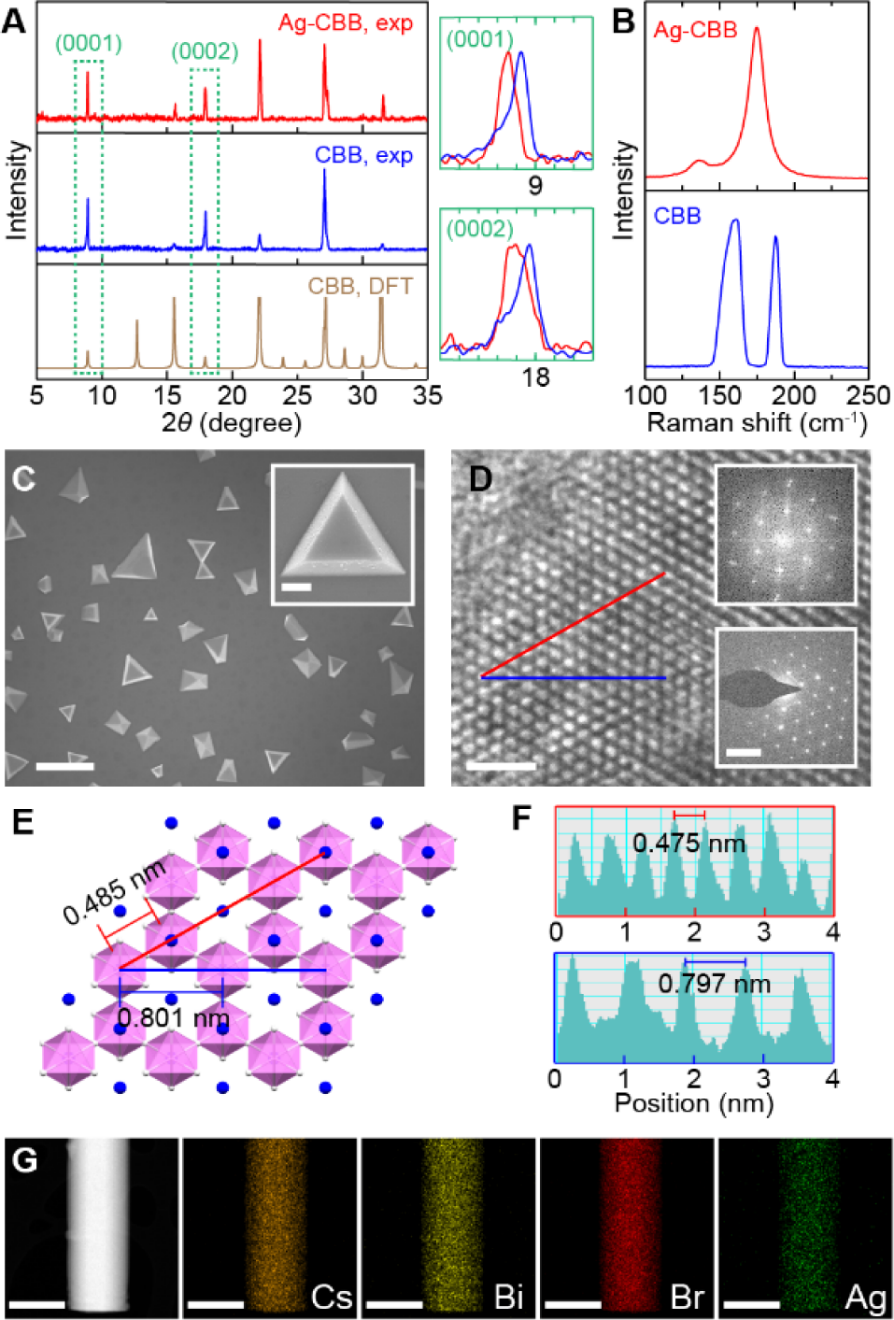
Structural analysis. (A) Measured and simulated XRD patterns. Insets on the right show magnified views of dashed green boxes. (B) Experimental Raman spectra of Ag-CBB (top) and CBB (bottom). (C) SEM image of Ag-CBB; scale bar, 10 μm. Inset scale bar, 2 μm. (D) TEM image of Ag-CBB, scale bar, 2 nm. Insets: FFT (top) and SAED (bottom) patterns; scale bar, 5 nm^–1^. (E,F) In-plane view of CBB (E) and projected bond distance analysis (F). (G) HAADF-TEM image and elemental maps for Cs, Bi, Br, and Ag; scale bar, 500 nm.

CBB and Ag-CBB microcrystals were synthesized using our home-built chemical vapor deposition system.^6^ The powder X-ray diffraction (XRD) patterns of as-grown CBB and Ag-CBB are shown in Figure 2A along with a DFT-simulated pattern. The overall crystal structure of Ag-CBB is expected to show little difference to that of CBB when a low concentration of Ag is introduced. A careful comparison of the two experimental patterns reveals good agreement in peak positions with no additional peaks resulting from other impurities, secondary phases, and unreacted precursors (Figure S2). We observe that the experimental relative intensities of the primary peaks at 8.9° and 17.9°, corresponding to (0001) and (0002) planes, respectively, are considerably greater than those in the simulated pattern. This indicates highly directional CVD growth along the stacking axis, which manifests as triangular crystal morphologies. Other XRD peaks observed at 15.4°, 22.1°, and 27° correspond to (1), (0), and (1) planes, and these planes are responsible for the presence of crystal morphologies with nontriangular symmetries (Figure S3). The measured interplane distance of 9.9 Å shows good agreement with the DFT-calculated unit cell height of 10.0 Å. Magnified views of the two primary (0001) and (0002) peaks show slight shifts toward smaller angles for Ag-CBB, indicating an expansion of interlayer spacing caused by the intercalation of Ag into the spacer regions of CBB.

We performed Raman spectroscopy to examine the chemical environment of Ag dopants. While bulk CBB is typically characterized by Raman vibrations from [BiBr_6_]^3−^ octahedra with Raman shifts of 164 and 190 cm^–1^,^29^ the Ag–Br vibrations at 139 and 178 cm^–1^ are known commonly to dominate the Raman response when both [AgBr_6_]^5−^ and [BiBr_6_]^3−^ are present together, as in Ag–Bi double perovskites.^30−32^ In undoped CBB, two clear peaks were observed at 164 and 190 cm^–1^, matching the signature vibrations of [BiBr_6_]^3−^ octahedra. Experimentally, we observe a distinct change in the Raman spectrum for Ag-CBB, with only two peaks at 139 and 178 cm^–1^, confirming that Ag ions indeed establish octahedral coordination with bromide anions (Figure 2B).

A scanning electron microscope (SEM) image of as-grown Ag-CBB is shown in Figure 2C. The Ag-CBB single microcrystals exhibit triangular morphologies, resulting from the hexagonal symmetry of CBB. The triangular morphology is observed for both CBB and Ag-CBB (Figures S4 and 2C). Atom-resolved transmission electron microscopy (TEM) imaging shows clear hexagonal symmetry, as is also revealed in the fast Fourier transform (FFT) image and selected-area electron diffraction (SAED) pattern (Figure 2D insets). A bond distance analysis was performed along the () and () directions (Figure 2F, shown by blue and red lines, respectively) in comparison to an expected atomic arrangement projected along the (0001) zone axis (Figure 2E). Experimentally calculated interatomic distances along these two major axes are 0.797 and 0.475 nm, respectively, which closely match with DFT-simulated values of 0.801 and 0.485 nm. Figure 2G presents a high-angle annular dark-field (HAADF) image, along with elemental distribution maps, obtained with energy dispersive X-ray spectroscopy (EDS) using scanning transmission electron microscopy (STEM). An elongated Ag-CBB nanorod was used for ease of transfer from the growth substrate to the TEM sample grid (Figure S5). We observe a uniform, homogeneous distribution of all four elements with no sign of phase segregation. Despite the homogeneity, Ag is expected to be randomly distributed throughout the crystals, making it challenging to ascertain the precise positions of individual Ag atoms. A quantitative stoichiometric EDS analysis reveals a 3:2 Cs:Bi atomic ratio with 1.8 ± 0.1% Ag incorporated, which corresponds to one Ag atom for every ∼59 unit cells (Figure S6).

There remain two possible scenarios regarding the position of Ag dopants in CBB: 1) an intercalation site in the spacer region and 2) substitution on a Bi site within the CBB layer. Each of these scenarios has the potential to account for the observed lattice expansion in XRD and the pronounced [AgBr_6_]^5−^ Raman response. For instance, the larger ionic radius of Ag^+^ (1.15 Å) compared to Bi^3+^ (1.03 Å)^33^ could result in expanded interlayer spacing through both intercalating and substitutional incorporation.

**Figure 3:**
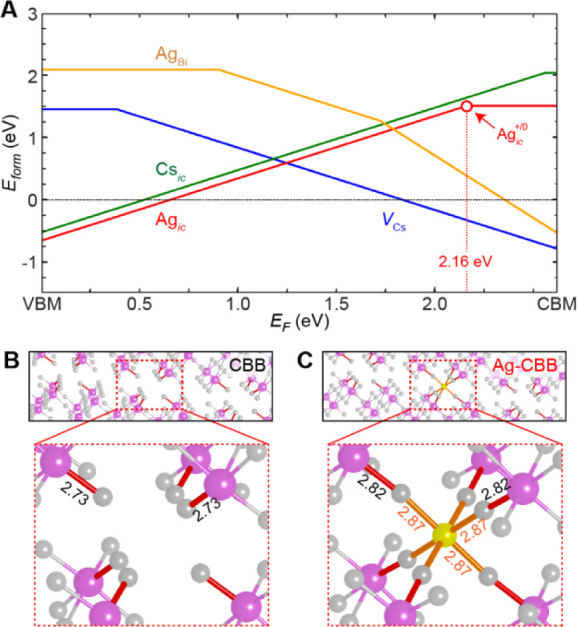
DFT analysis of defects. (A) *E*_form_ of various defects in CBB calculated as a function of *E_F_* above the VBM. Red circle denotes the  transition point 2.16 eV above the VBM. (B,C) Calculated bond environment around an intercalation site before (B) and after (C) Ag intercalation. Insets: Magnified views with selected bond lengths in Å.

We address this question by performing a first-principles DFT analysis for different sites of Ag incorporation. Figure 3A presents calculated energies of formation (*E*_form_) as a function of Fermi energy (*E_F_*) above the valence band maximum (VBM) for select types of Ag impurities and native defects; among them, intercalating Ag (Ag*_ic_*), Ag substituting Bi (Ag_Bi_), intercalating Cs (Cs*_ic_*), and native Cs vacancies (*V*_Cs_). *E*_form_ represents an energetic penalty required for the formation of each type of defects. In principle, interstitials Ag_*i*_ and Cs_*i*_ may also form within the interstitial sites in CBB layers; however, cation interstitials have high formation energies in other halide perovskites,^34^ so we expect them not to be relevant. Ag substituting for Cs (Ag_Cs_) is also relatively high in energy and lacks the charge state transition levels relevant for emission, so we do not discuss it here. For ease of presentation, we focus on intermediate chemical potential conditions (Table S1), although other choices of chemical potential generally lead to similar results (Figures S9 and 10).

As depicted in Figure 3A, intercalated  will be the lowest energy Ag species for most positions of *E_F_*; additionally, it may be lower in energy than the lowest-energy native donor defect, . Ag_Bi_, which is an electron acceptor for most of the band gap ( and ), is significantly higher in energy and will be present in undetectable quantities for most chemical potentials (see Table S2). Only under extreme Bi-poor/Br-rich chemical potentials does  become more energetically favorable than . It follows, therefore, that we expect Ag to fill interlayer vacancy sites as  when it is present. The positive charge of Ag_*ic*_^+^ is balanced by equal concentrations of *V*_Cs_^−^, pinning *E_F_* approximately 1.25 eV above the VBM (at the intersection of the red and blue lines in Figure 3A).

**Figure 4:**
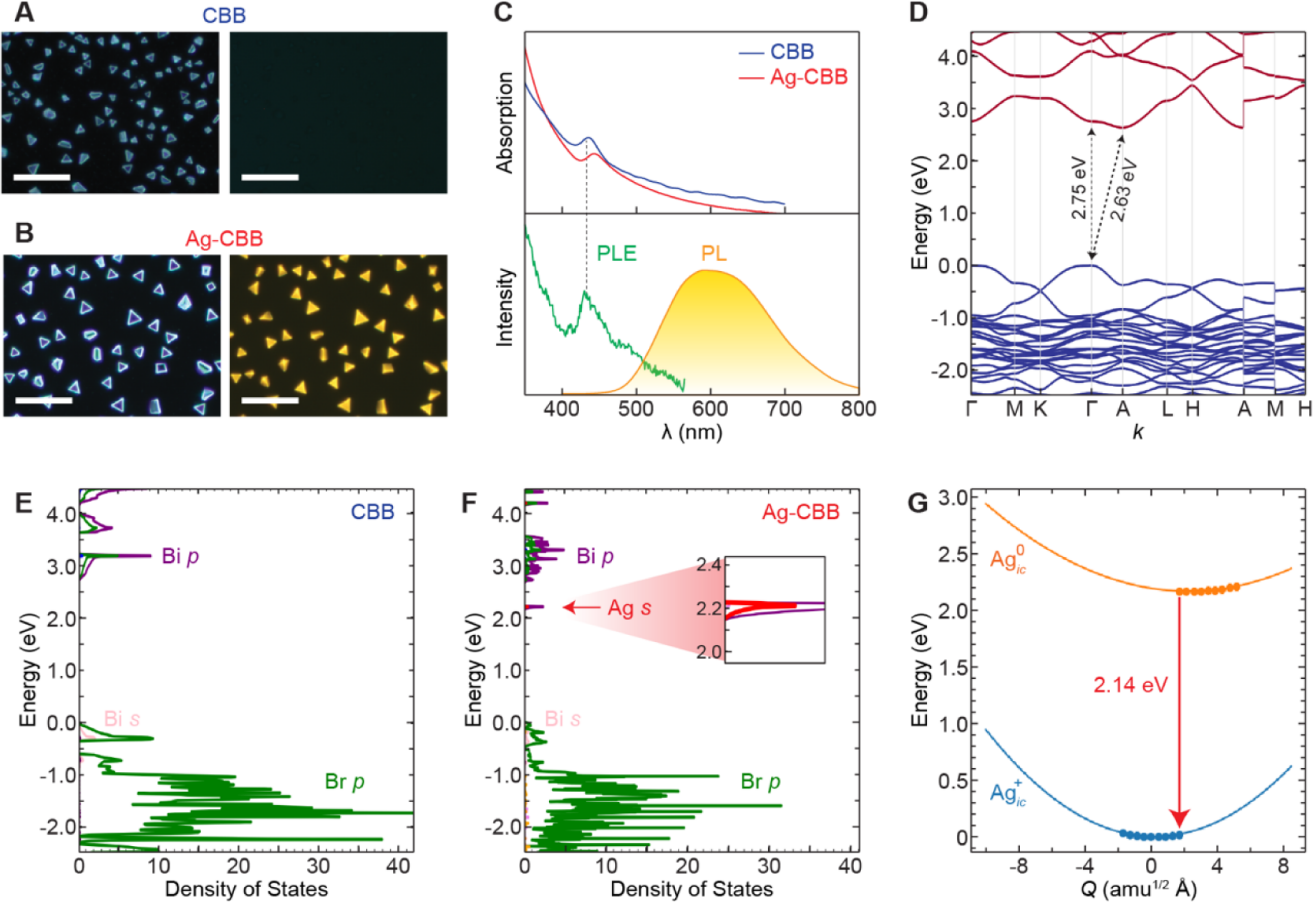
Optical analysis. (A,B) DF (left) and PL (right) images of CBB (A) and Ag-CBB (B); all scale bars, 40 μm. (C) Measured absorption (top), and PL and PLE spectra of Ag-CBB (bottom). (D) Calculated band structure of CBB. (E,F) Calculated PDOS of CBB (E) and Ag-CBB (F). Inset shows magnified view on Ag *s* peak. (G) Configuration coordinate diagram for Ag_ic_.

As mentioned previously,  adopts an octahedral configuration with nearby Br atoms. The [AgBr_6_]^5−^ octahedra exhibit equilateral bond lengths of 2.87 Å for every Ag–Br bond. The presence of Ag causes a ∼3.4% bond extension of the Bi–Br bonds pointing toward the Ag intercalation site, from 2.73 Å without Ag present (Figure 3B) to 2.82 Å with Ag present (Figure 3C). This bond distortion in the [BiBr_6_]^3−^ octahedra leads to the formation of an electron trap state in the form of Bi 6*p* states hybridizing with Ag 5*s* states, which is present slightly below the conduction band minimum (CBM) as presented in Figure 4F. This hybridized, localized state is attributed to the  charge transition level lying 2.16 eV above the VBM when it captures an electron, as denoted by a red circle in Figure 3A. Based on the position of the  transition level, we hypothesize that this state is responsible for the yellow emission observed in Ag-CCB.

Notably, Cs*_ic_* is not a candidate for yellow emission, despite also forming favorably, primarily in the +1 charge state, and in the same crystallographic site as Ag*_ic_*. This difference can be attributed to Cs having a significantly higher ionization energy than Ag (3.89 eV for Cs, 7.58 eV for Ag),^35^ meaning that the  transition level will be significantly higher in the band gap. This distinction hints that transition metals, such as Ag, are optimal dopants for introducing emission in layered materials like CBB, while alkali and alkaline earth metals are likely unsuitable for that purpose.

### Optical Analysis

We subsequently investigated the optical properties of Ag-CBB. Figure 4A and B present optical dark-field (DF) and PL images of CBB and Ag-CBB, showing their characteristic triangular morphologies. When excited by a UV flashlight, no measurable amount of PL emission was detected from CBB (Figure 4A), which is consistent with near-dark RT PL reported in existing literature.^10,14^ On the other hand, a bright and vivid yellow emission was observed from Ag-CBB at RT (Figure 4B). Absorption, PL, and photoluminescence excitation (PLE) spectra of Ag-CBB are plotted in Figure 4C, along with an absorption spectrum of CBB. The host CBB has a direct bandgap  and a slightly lower-lying indirect transition at . For comparison, our DFT-calculated bandgaps are  (Γ) and  (Γ → A) (Figure 4D). Due to the small difference between the energy gaps of direct and indirect transitions, absorption behavior is dominated by the direct transition, as represented by a single absorption peak at 436 nm. The absorption profiles of our CBB and Ag-CBB are mostly similar, with a small shift of the band-edge absorption peak position from 436 to 441 nm. Experimentally derived direct bandgaps (Figure S7) are 2.62 and 2.55 eV for CBB and Ag-CBB, respectively, which are in qualitative agreement, implying that Ag intercalation does not significantly change the band structure of the host crystal.

The PL curve in Figure 4C reveals a broad spectrum with a peak position at ∼600 nm with a color coordinate of (0.5005, 0.4738) (Figure S8), a full-width-at-half-maximum (fwhm) of ∼160 nm, and a Stokes shift of ∼0.75 eV. Noting that  is a polaronic donor, we highlight several important features characteristic to PL of the impurity-trapped small polarons.^36−38^ First of all, the large line width suggests that the emission of Ag-CBB does not exhibit the characteristics of emission from tightly bound excitons or atomic transitions. Instead, the radiative transition involves a strong exciton–phonon interaction, as commonly observed in STEs^39^ and for indirect transitions.^40^ Also, such a large Stokes shift indicates that the radiative transition does not originate directly from free exciton (FE) states near the band edges, but rather involves energy/charge transfer to a distinct deep state from which the emission takes place. Lastly, the emission band is primarily observed only with excitation energies at or greater than the bandgap energy, which implies an absence of a direct excitation pathway for 600 nm emission. The experimental PLE spectrum shows a single peak centered at 420 nm, a wavelength that corresponds to the direct bandgap absorption of the host CBB. The absence of additional PLE peaks in the spectral vicinity of the emission peak (600 nm) indicates the absence of a direct photoexcitation pathway for this emission.

The partial densities of states (PDOS) for CBB and Ag-CBB are presented in Figure 4E,F. Unlike CBB, which exhibits no midgap state (Figure 4E), Ag-CBB possesses a highly localized  state near the CBM (Figure 4F), confirming the nature of the  charge transition level shown in Figure 3A. The  transition represents the change of the oxidation state of Ag after it captures an electron and therefore can describe electron transfer from the CBM to .

We constructed a configuration coordinate diagram to validate the role of Ag_ic_ in electron capture (Figure 4G). With  and  regarded as the ground and excited states, respectively (), the excited state will release an electron with an emission energy of 2.14 eV to return to the ground state. We find a coordinate shift (Δ*Q*) of 1.72 Å amu^1/2^ with the Franck–Condon relaxation energies of 26.8 and 15.2 meV in the ground and excited states, respectively. The small Δ*Q* reflects that the configurations of  and  are nearly isostructural, such that little energy is lost in structural relaxations, a feature distinct from STEs that require nontrivial relaxation energies. While 2.14 eV is slightly higher than the observed yellow emission, our configuration coordinate diagrams slightly overestimate emission energies,^41^ so the agreement is adequate. Based on the charge state transition levels for other relevant defects (e.g., , , and ; Figures 3A and S6), it is unlikely that any of them could be responsible for yellow emission.

**Figure 5:**
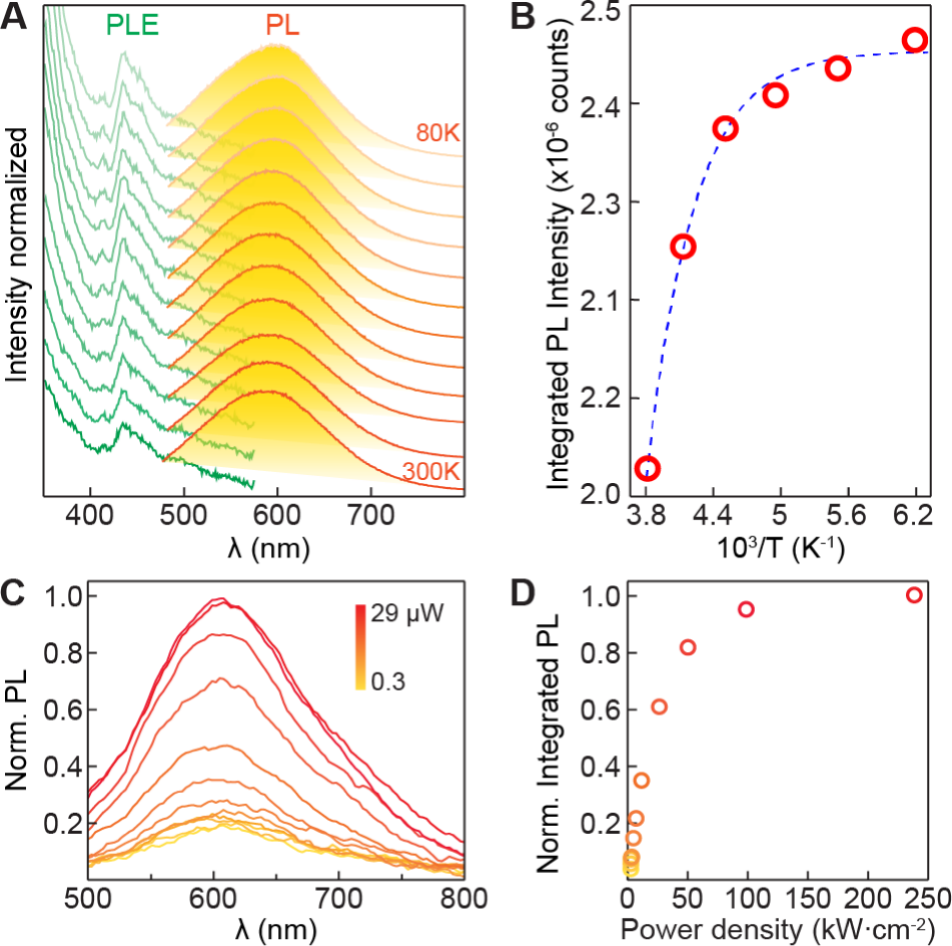
Temperature- and power-dependent PL. (A) Temperature-dependent PL and PLE of Ag-CBB measured at 300–80 K with 20 K increment. Plots are vertically offset for clarity. (B) Integrated PL intensity against inverse temperature with fit to Arrhenius function. (C) Normalized PL spectra measured with the excitation power varying from 0.3 to 29 μW. (D) Normalized integrated PL intensity as a function of excitation power density.

The temperature-dependent emission behavior is shown in Figure 5. No significant change in spectral features was observed from both PL and PLE when measured at 300 K down to 80 K. This means that the PL of Ag-CBB does not significantly involve phonons and therefore is not heavily affected by temperature. This is particularly the case because Ag is an extrinsic impurity that causes a permanent structural distortion, as opposed to a transient distortion that accompanies dynamic lattice motions for STEs, thus the emission is less sensitive to temperature. An overall increase in the PL intensity was observed with decreasing temperature. We show an integrated PL intensity profile as a function of inverse temperature (Figure 5B) with a fit to an Arrhenius function to obtain the exciton binding/dissociation energy (*E*_b_) of 205 ± 15 meV. This *E*_b_ is lower than a reported *E*_b_ of 322 meV for CBB,^30^ an indication of a reduced electrostatic attraction between the electrons and holes.

The relationship between excitation power and PL intensity is examined. While the intensity of FE emission linearly increases with an increasing excitation power, emission involving an energy transfer to dopants typically exhibits a sublinear saturation behavior as the number of available dopant sites decreases at high power.^42,43^ We have measured power-dependent PL of Ag-CBB using a confocal laser microscope by varying an excitation power from 0.3 to 29 μW (Figure 5C). A 405 nm excitation laser beam was focused into a diffraction-limited spot size and rapidly scanned through sample crystals with ∼3.2 μs dwell time at each position. An increase of the overall size of the PL envelop was observed with no apparent change in the spectral shape, indicating an absence of additional photophysical processes within the measurement conditions. The integrated areas of PL spectra are plotted as a function of excitation power density in Figure 5D. Initially, the integrated PL intensity showed a linear dependence at low power densities below approximately 25 kW cm^*–*2^. As the excitation power density increased, the Ag emission entered a sublinear regime, and eventually saturated at high power densities.

Following these results, we introduce the concept of intercalation-mediated bound interlayer excitons (BIEs), as formed in our layered Ag-CBB crystals. A BIE describes a localized pair of photocarriers separated in different layers, with electrons polaronically trapped by intercalating Ag and holes left behind in the CBB layers. This notion captures two distinct characters of bound excitons (BEs) and interlayer excitons (IEs) at the same time. BEs describe excitons bound to chemical impurities, such as vacancies, dopants, and surface adsorbents.^42^ Their PL is known to exhibit greater oscillation strengths with emission energies lower than PL from FEs due to the energy lost during the trap processes, as has been demonstrated in doped III–V semiconductors^44−46^ and atomically thin transition metal dichalcogenides with natural vacancies.^47−50^ These behaviors were observed in Ag-CBB from an enhanced PL intensity, large Stokes shift, and calculated trapping energy presented in Figure 4. On the other hand, IEs are observed in 2D heterostructures,^51,52^ where the dissociation of excitons takes place due to the type-II bandgap alignment of 2D layers stacked atop each other.^53^ Spatial localization of IEs has been demonstrated by several distinct mechanisms, such as periodic Moiré potentials in twisted bilayers^54^ and coupling of IEs with characteristic lattice phonons.^55,56^ Our BIEs are bound to chemical Ag dopants present in the interlayer void spaces, thereby exhibiting both bound and interlayer characters. The out-of-plane distance between Ag and the CBB layer in Ag-CBB—representative of e–h separation—is only a half a unit cell (∼5 Å), which is distinctly shorter than the typical separation distances observed in 2D heterobilayers.^57,58^ As such, we anticipate that the BIEs in Ag-CBB will exhibit unique exciton behaviors, and presumably a high luminescence efficiency and moderately prolonged decay times.

**Figure 6:**
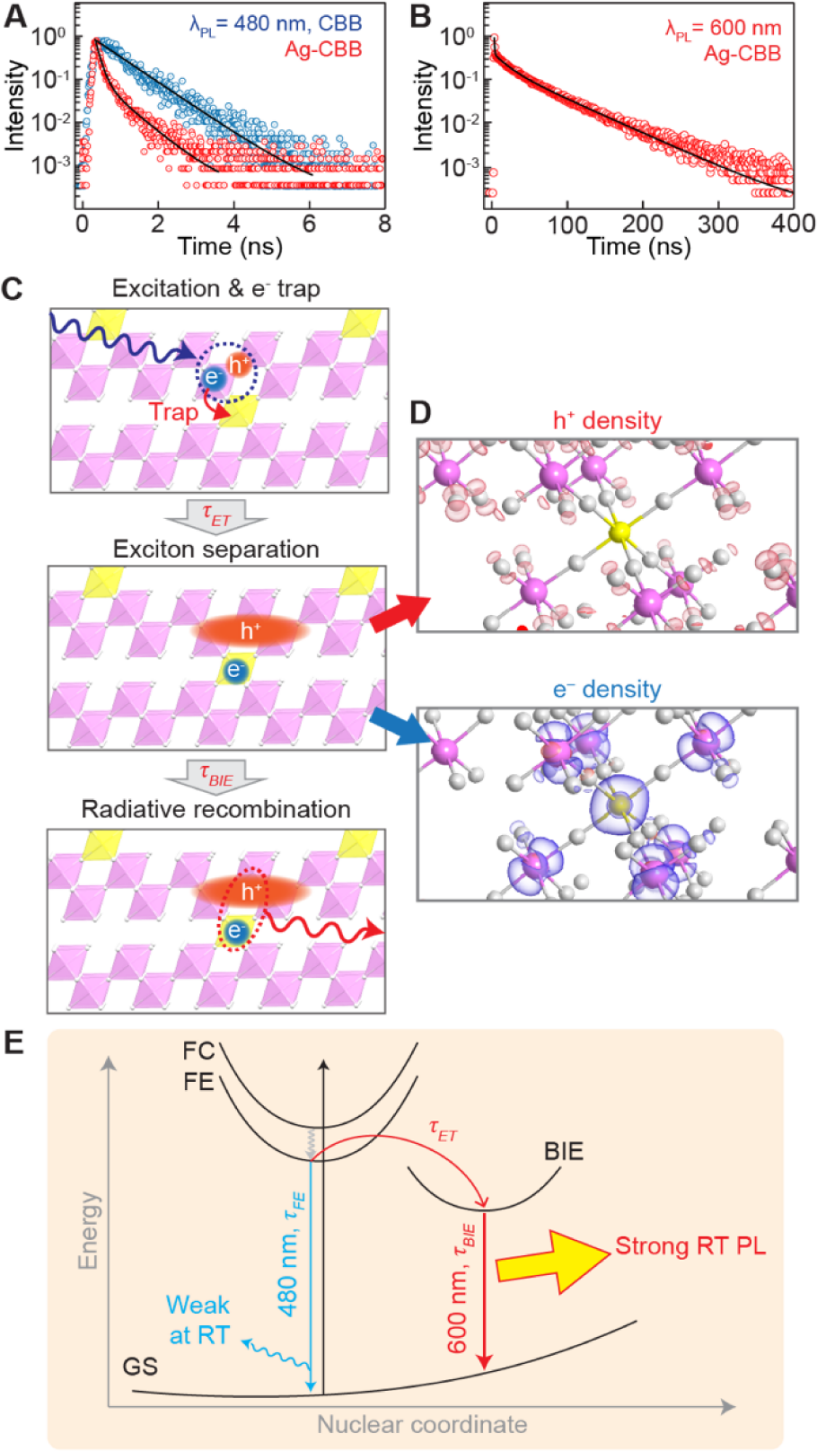
Dynamic behavior of BIE. (A,B) Time-resolved PL spectra measured at 480 (A) and 600 nm (B). (C) Stepwise illustration of BIE process. (D) Charge density isosurfaces of holes (top, red) and electrons (bottom, blue). Cs atoms are omitted for clarity in (C) and (D). (E) Energy diagram of Ag-CBB. FC: free carriers, FE: free excitons, BIE: bound interlayer excitons, GS: ground state, τ_FE_: FE decay time, τ_BIE_: BIE decay time, and τ_ET_: electron trapping time constant.

Time-resolved PL decay measurements were performed to examine the decay dynamics of these BIEs in Ag-CBB. Although PL was not initially observed from undoped CBB in our imaging microscope under incoherent excitation (Figure 4A), a measurable amount of PL was detected at 480 nm (corresponding to the bandgap) using coherent picosecond-pulsed laser excitation after an hour-long acquisition. Pristine CBB exhibits a single decay time (τ_FE_) of 1.1 ns from the radiative recombination of FEs (Figure 6A). For Ag-CBB at the same detection wavelength, we observed two processes with shorter decay times of 0.13 and 0.69 ns. The acceleration of FE emission is attributed to greater nonradiative contributions caused by lattice distortions and charge trapping. The determination of the electron-trapping time (τ_ET_) in the current work is limited by the instrument response function (∼40 ps), which is not sufficiently short, and an ultrafast measurement modality is necessary for an accurate quantification.

A PL decay curve of the 600 nm emission of Ag-CBB is presented in Figure 6B. We observe a substantial increase in decay time (τ_BIE_) from subnanosecond to 38.6 ns, a factor of 35 increase compared to the 1.1 ns τ_FE_ of undoped CBB. We attribute this prolonged lifetime to the spatial separation of photocarriers. A stepwise illustration of the motion BIEs is presented in Figure 6C. Initially, FEs are photogenerated in a CBB layer, followed by a quick trapping of the electron by . The dissociation of electrons and holes, which forms an BIE, reduces the electrostatic interaction between the charge carriers, increasing their lifetime. The calculated charge density isosurfaces of electrons and holes at this stage are displayed in Figure 6D, clearly depicting electron clouds localized on the Ag atom, while holes are spatially distributed within CBB layers. Eventually, the charge carriers radiatively recombine to emit photons after τ_BIE_. A schematic energy diagram is presented in Figure 6E, summarizing all the carrier dynamics discussed above.

## Discussion

In conclusion, we have demonstrated the successful intercalation of Ag into CBB single crystals through controlled CVD synthesis. The  dopants intercalate into the ordered vacancy planes with octahedral coordination to surrounding Br atoms, forming an optically active trap state hybridized with neighboring Bi cations. The Ag-intercalation creates spatially separated BIEs upon photoexcitation due to the strong electron-trapping character of , with electrons trapped by interlayer Ag and holes remaining in CBB layers. Radiative recombination of BIEs exhibits a bright emission at 600 nm at RT with a 38.6 ns decay time, a factor of 35 longer than FE decays. The BIE, as a new form of IEs, achieved here through crystalline intercalation of Ag^+^ into CBB and related layered materials, is expected to inspire new research directions toward the development of future optoelectronic devices based on CBB and related layered materials.

## Experimental Section

### Materials

Bismuth bromide (99.9%), silver bromide (99.99%), cesium bromide (99.99%), acetone (≥ 99.5%), and isopropanol (≥99.5%) were purchased from Sigma-Aldrich. All chemicals were used as purchased without further purification.

### CVD Synthesis of CBB and Ag-CBB

CBB and Ag-CBB were grown on a SiO_2_ wafer (Nova Electronic Materials; *n*-type (100) Si with 3000 Å thermal oxide; 1–10 Ω cm) and on microscope glass slides cut in a size of 1 cm × 2 cm in our home-built CVD system.^6^ Growth substrates were first cleaned by sequentially sonicated in acetone and isopropanol, blow-dried with nitrogen gas, treated in a UV/O_3_ cleaner (Samco UV-1) at 150 °C for 5 min, and placed 12 cm downstream from the furnace center. BiBr_3_, AgBr, and CsBr powders were placed in a quartz source boat at the center of a single-zone furnace (Fisher Scientific; Lindberg/Blue M Mini-Mite). Prior to the reaction, the quartz tube was baked at 950 °C for 1 h with 50 sccm Ar gas under vacuum and was allowed to cool to room temperature. After loading growth substrates and precursors, a controlled experiment was performed at 500 °C at 150 Torr pressure with a constant 70 sccm Ar flow. The temperature was increased at a 40 °C min^–1^ ramp rate to the desired reaction temperature. The reaction was allowed to run for 30 min and allowed to cool naturally to 100 °C before opening the furnace, while the pressure and Ar flow were maintained throughout. Nanowires of the Ag-CBB were grown by changing the reaction conditions, maintaining the hexagonal symmetry but resulting in elongation along one direction. These nanowires were used for STEM mapping and analysis.

### Electron Microscopy

SEM images were acquired using a JEOL 7500F microscope with a cold field emission emitter in the secondary electron detection mode, operating at a 15.0 kV accelerating voltage and an emission current of 10 μA. The JEOL 7500F was equipped with an Oxford EDS system, which was used for elemental analysis and mapping. TEM was performed using a Thermo Fisher Talos F200X G2 instrument. An accelerating voltage of 200 kV was used for the imaging. The CTEM point resolution was 0.25 nm, with a line resolution 0.12 nm and an information limit < 0.12 nm with 0.10 nm attainable. Lacey Carbon, 300 mesh, gold, with an approximate grid hole size of 63 μm grid was used for the TEM and HAADF measurements.

### Powder X-Ray Diffraction

Powder X-ray diffraction (pXRD) was carried out on a Bruker D8 advance diffractometer using CuKα radiation (λ = 1.5418 Å) having a LYNXEYE (one-dimensional (1D) mode) detector over the 2θ range of 5–50° with a step size of 0.02° for 15 min.

### Raman Spectroscopy

Raman spectra were recorded at room temperature on a HR-800 Raman spectrophotometer (Jobin Yvon, Horiba, France) using monochromatic radiation emitted by a He–Ne laser (532 nm), operating at 50 mW and with a spectral resolution of 0.3 cm^–1^. An objective of ×50 magnification was used both to focus and to collect the scattered light from the CBB and Ag-CBB crystals deposited on Si wafers.

### Optical Imaging and PL Spectroscopy

Absorption spectra were collected using an Evolution 220 ultraviolet–visible (UV–Vis) spectrometer (Fisher Scientific) containing dual silicon photodiodes. The sample was grown on a glass substrate to measure the absorption, and clean glass was used as a blank. The sample absorption spectrum was measured from 300 to 700 nm with an integration time of 0.30 s and a scan speed of 200 nm/min. Optical images were captured using a Zeiss upright optical microscope (Axio Imager A2m). Dark-field (DF) images were acquired using micro-LED light, and PL images were taken using a band filter of 450–490 nm for excitation and a long-pass filter at 515 nm for emission (ZEISS filter set 09). Static PL spectra were collected by fiber-coupling PL emission from the microscope to a grating spectrograph (Horiba iHR550) interfaced with a liquid nitrogen-cooled CCD detector (Horiba Symphony II).

### Variable-Temperature PL

Temperature-dependent PL and PLE were measured using an Edinburgh FLS1000 photoluminescence spectrometer attached to an Optistat DN cryostat equipped with a Mercury iTC temperature controller (Oxford Instruments). The sample was excited by using a xenon lamp, and the emission and excitation was collected from 465 to 800 nm and 350 to 565 nm, respectively.

### Time-Resolved PL Spectroscopy

Time-resolved photoluminescence (TRPL) measurements at 480 nm for CBB and Ag-CBB were performed using a diode-pumped Nd:YVO_4_ laser (Spectra Physics Vanguard) as a source laser, which produces 2.5 W average power at 532 and 355 nm, with 13 ps pulses at a 80 MHz repetition rate. The output from this laser excites a synchronously pumped cavity-dumped dye laser (Coherent 701–3), producing 5 ps pulses at 800 nm (LDS-751 dye, Exciton) at a repetition rate of 4 MHz. The 800 nm output pulses were frequency-doubled using a Type-I LiIO_3_ crystal to produce 400 nm pulses at the same repetition rate. Emission was collected using a reflecting microscope objective, and the wavelength was selected using subtractive double monochromators (Spectral Products CM112), equipped with microchannel plate PMT detectors (Hamamatsu R3809U-50). The detection electronics (Becker and Hickl SPC 132) provided an instrument response function of ca. 40 ps, and the instrument was controlled using software written in-house. The lifetime at 600 nm for Ag-CBB was measured using a time-correlated single-photon counting (TCSPC) setup. The data acquisition card (DAQ) is from Edinburgh Instruments (TCC900). The laser used for the experiment is a 405 nm pulsed laser from Picoquant (LDH-D-C-405M, CW-80 MHz). The detector is a photomultiplier tube (PMT) from Hamamatsu (H7422-40).

### Power-Dependent PL

Power-dependent PL was measured using a Leica Stellaris 5 confocal microscope with a Leica 63× HC PL APO OIL (1.40 NA) objective lens at the Center for Advanced Microscopy at MSU. The sample was excited with a 405 nm continuous wave laser and emission was collected between 500 and 800 nm using a HyD S2 detector. The pixel dwell time was set to 3.1625 μs, and the scan speed was 400 Hz. A series of spectral images was acquired with 10 nm bandwidth and 5 nm step size. The laser spot size was determined using the theoretical XY optical resolution (d_*xy*_ = 0.61λ/NA). Laser power was measured after the objective lens using a photodiode detector (918D-SL, Newport) with an optical power meter (1936-R, Newport).

### DFT Calculations

First-principles calculations based on DFT were conducted using the Heyd, Scuseria, and Ernzerhof (HSE06) screened hybrid functional,^59^ as implemented in the Vienna *Ab initio* Simulation Package (VASP).^60^ The HSE06 mixing parameter, which quantifies the fraction of nonlocal Hartree–Fock exchange included, was set to 28%, which leads to good agreement with the prior experimentally reported direct band gap of ordered CBB.^14^ Core electrons were represented with projector-augmented wave potentials,^61^ with the Cs 5*s*^2^ 5*d*^6^ 6*s*^1^, Bi 6*s*^2^ 6*d*^3^, Br 4*s*^2^ 4*d*^5^, and Ag 5*s*^1^ 4*d*^10^ electrons treated explicitly as valence states. Spin–orbit coupling was included due to the presence of heavy elements such as Bi. A plane wave energy cutoff of 500 eV was used for all calculations. Total energies were converged to within 10^–5^ eV, and forces were considered converged when less than 10 meV/Å. The 14-atom unit cell (containing one formula unit) of ordered CBB was converged using a 4 × 4 × 4 Γ-centered **k**-point mesh. Defect formation energy calculations were conducted in a 2 × 2 × 2 supercell, containing 104 atoms, which was relaxed using only the Γ **k**-point. Defects were calculated for both the ordered structure, which is stable at room temperature, and a low-symmetry, disordered structure, which is 50 meV per formula unit lower in energy than the ordered cell at 0 K; comparative results between these two structures can be found in the Supporting Information. More details regarding defect formation calculations, including our treatment of chemical potential conditions, are provided in the Supporting Information. Images of atomic environments were generated using the VESTA3 software.^62^

## Supporting Information Available

Additional experimental and calculation results and detailed DFT calculation methods (PDF)
