# Supplementary material for: Ag Intercalation in Layered Cs3Bi2Br9 Perovskite for Enhanced Light Emission with Bound Interlayer Excitons
Source: J Am Chem Soc. 2024 Jul 10;146(29):19919–28. doi: 10.1021/jacs.4c03191 (PMC11273344; doi:10.1021/jacs.4c03191)
Supplement: Supplementary file 1 — ja4c03191_si_001.pdf [file ja4c03191_si_001.pdf]

Supporting Information for

Ag Intercalation in Layered $Cs_3Bi_2Br_9$ Perovskite for Enhanced Light Emission with Bound Interlayer Excitons

Anupam Biswas[1†], Andrew J. E. Rowberg[2†], Pushpender Yadav[1], Kyeongdeuk Moon[1], Gary J. Blanchard[1], Kyoung E. Kweon[2], Seokhyoung Kim[1]*

[1]Department of Chemistry, Michigan State University, East Lansing, MI 48824, USA

[2]Quantum Simulations Group and Laboratory for Energy Applications for the Future (LEAF), Lawrence Livermore National Laboratory, Livermore, CA 94550, USA.

Table of Contents:

1. Additional supplementary data

Figure S1: Crystal structure. Unit cell of CBB with different orientations.

Figure S2: X-ray diffraction comparison with precursors.

Figure S3: Visualization of crystallographic planes.

Figure S4: Morphology and composition of CBB

Figure S5: Morphology of Ag-CBB nanowires (NWs)

Figure S6: EDS spectrum of Ag-CBB.

Figure S7: Band gap measurements.

Figure S8: CIE coordinate of Ag-CBB.

1.1. Exciton binding energy

2. Detailed DFT calculation method

2.1. Defect formation energies

2.2. Chemical potentials

Figure S9. Thermodynamic stability of CBB.

Table S1. Chemical potential conditions for CBB.

Figure S10. Formation energies of native point defects and silver impurities under a range of chemical potentials for disordered CBB.

Table S2. Concentrations of silver species in disordered CBB.

1. Additional supplementary data

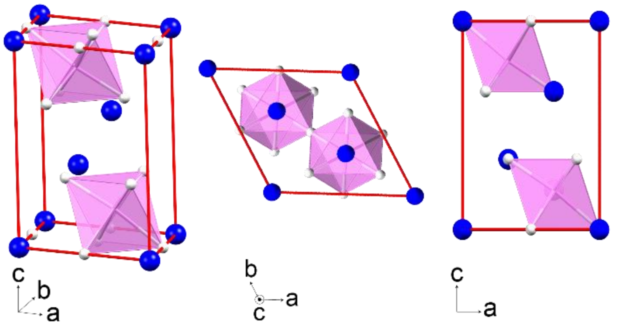

Figure S1: Crystal structure. Unit cell of CBB with different orientations.

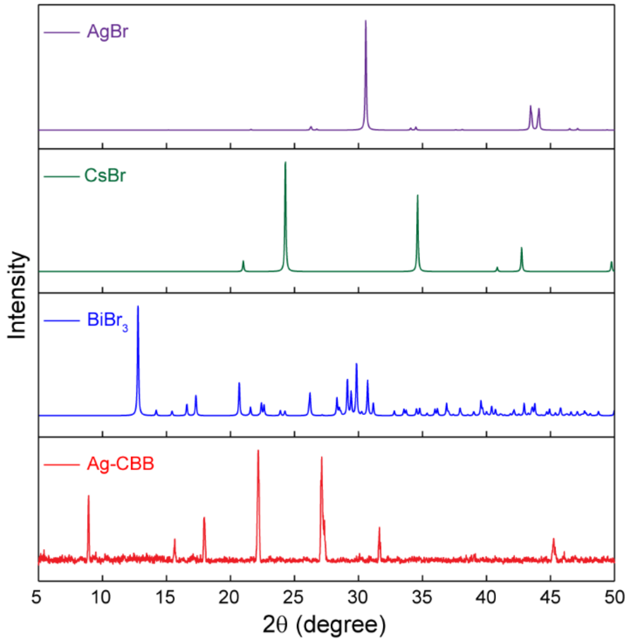

Figure S2: X-ray diffraction comparison with precursors. Comparison of experimental Ag-CBB XRD with $BiBr_3$, CsBr, and AgBr precursors show no presence of unreacted components in the synthesized crystals.

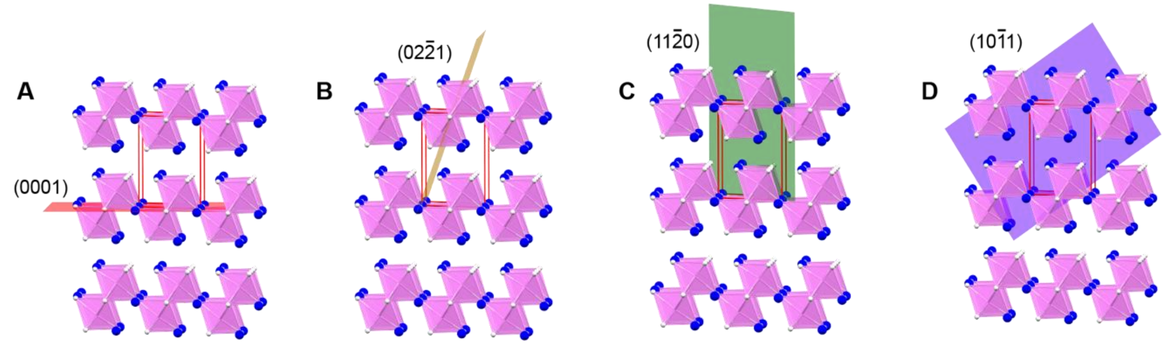

Figure S3: Visualization of (A) (0001), (B) (02$\bar{2}$1), (C) (11$\bar{2}$0), and (D) (10$\bar{1}$1) planes.

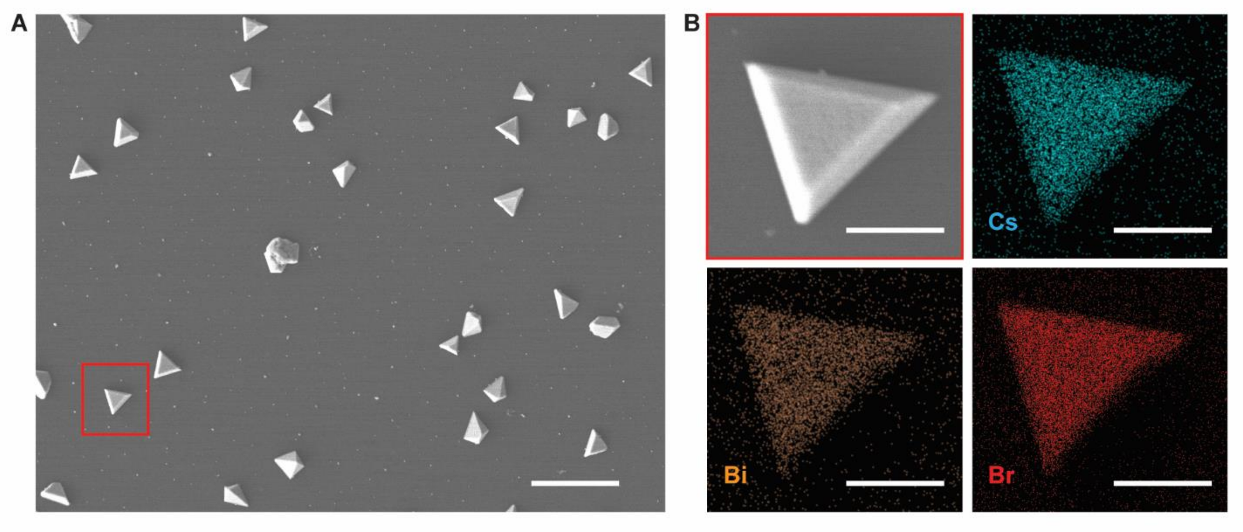

Figure S4: Morphology and composition of CBB. SEM image and elemental maps of undoped CBB. Scale bars: 30 μm (SEM image) and 5 μm (inset and elemental maps). The red square in the SEM image shows the selected particle used for mapping, which displays a homogeneous distribution of the elements.

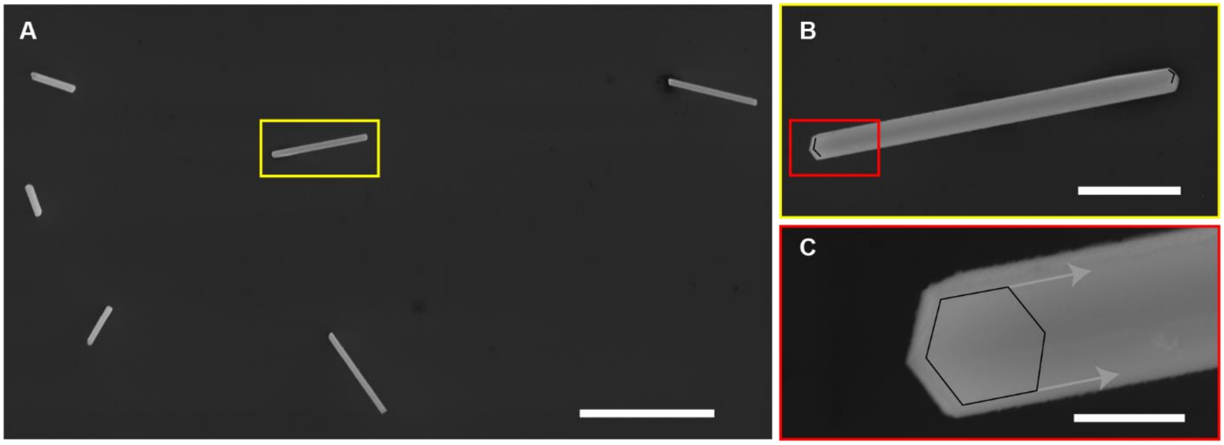

Figure S5: Morphology of Ag-CBB nanowires (NWs): SEM images of Ag-CBB; scale bar, 25 μm (A), 5 μm (B) and 1 μm (C). The magnified images from A in panel C show the edge of the NWs having hexagonal symmetry with elongation by giving extended reaction time, giving rise to the NW morphology.

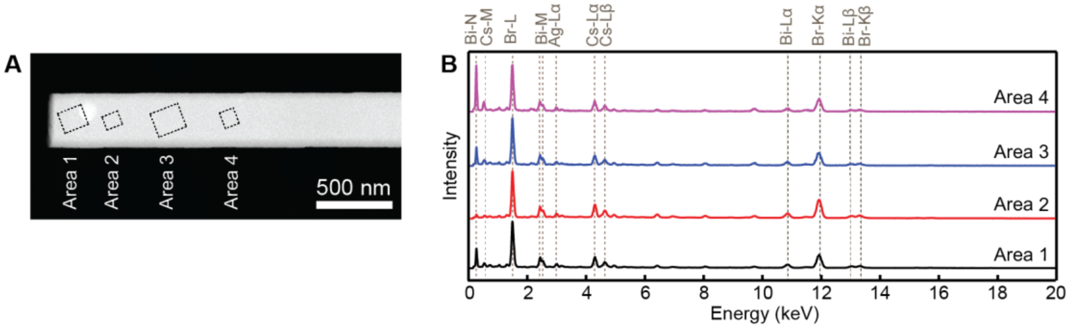

Figure S6: EDS of Ag-CBB. (A) HAADF images of the nanorod particle including the four different areas used for EDS spectra collection. (B) EDS spectra of the above-mentioned areas. All the spectra show the presence of a Ag-Lα line at 2.9 keV.

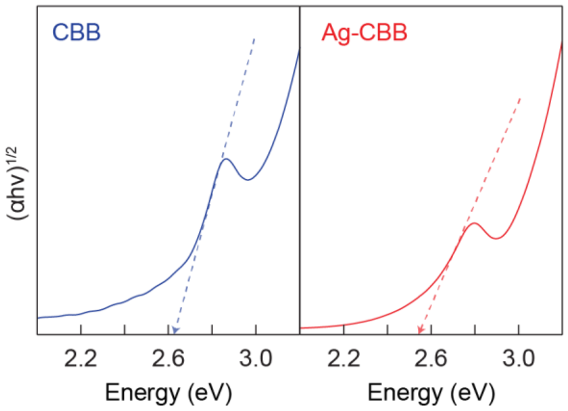

Figure S7: Band gap measurements. Tauc plots of CBB and Ag-CBB showing obtained band gaps of 2.62 eV and 2.55 eV, respectively.

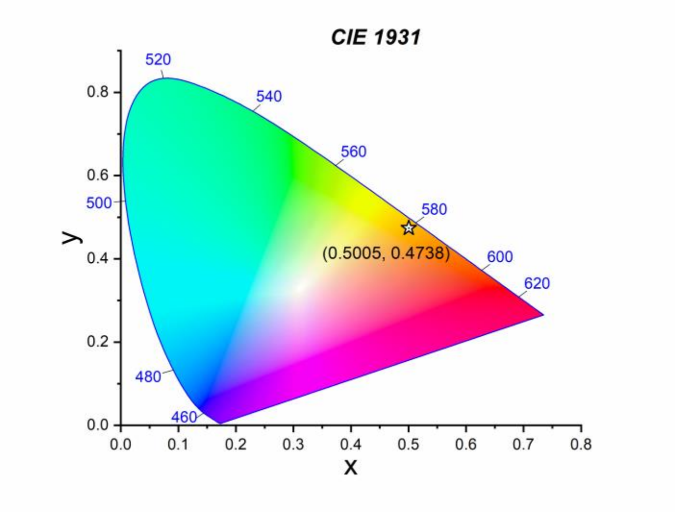

Figure S8: CIE coordinate of Ag-CBB emission.

1.1. Exciton binding energy.

We used the following Arrhenius function to calculate the exciton binding energy of the Ag-CBB sample:

$$I\ (T) = I(0)/(1 + A\ \exp^{(E_b/k_\mathrm{B}T)})$$

$I(T)$ and $I(0)$ are the integrated photoluminescence intensity at a given temperature, T, and 0 K, respectively, E_b is the exciton binding energy, and k_B is the Boltzmann constant.

2. Detailed DFT calculation method

2.1. Defect formation energies.

Defect formation energies E_form are calculated using the equation[1]:

$$E_\mathrm{form}(D^q) = E(D^q) - E_\mathrm{bulk} + \sum n_i\mu_i + qE_F + \Delta^q.$$

Here, D^q represents a defect in charge state q. $E(D^q)$ is the total energy of a supercell containing D^q, and E_bulk is the total energy of the pristine (defect-free) supercell. $|n_i|$ is the number of atoms of species i removed $(n_i > 0)$ or added $(n_i < 0)$ from the supercell to create the defect, and μ_i are the corresponding chemical potentials. E_F is the position of the Fermi level, relative to the valence band maximum (VBM), and Δ^q is a finite-size correction term to account for charge interactions with repeated images across the periodic boundaries.[2-3]

E_form is exponentially related to the defect concentration c as follows:

$$c(D^q) = N_{\text{sites}} \exp\left(-\frac{E_{\text{form}}(D^q)}{k_{\text{B}}T}\right).$$

N_{sites} is the site-concentration of defects in the unit cell, k_{B} is Boltzmann's constant, and T is the temperature. It follows that lower formation energies lead to exponentially higher concentrations, so the chemistry of the system will largely be unaffected by high-energy defects.

When calculating defect formation energies initially, we treat E_F as a free variable, meaning that formation energies are plotted as lines with slope q on the axes E_F-*vs*-E_{form}. However, the actual E_F will be determined by the requirement that the overall system must be charge neutral. To a good approximation, E_F will thus be located at the intersection point of the lowest-energy positively and negatively charged defects. For Ag-CBB, under most relevant chemical potential conditions, the positive charge of each Ag_{ic}^{+} is balanced by the negative charge of V_{Cs}^{-}, thereby positioning E_F approximately 1.25 eV above the VBM at the intersection of the red and blue curves in Fig. 3. This implies that the probable chemical formulation of Ag-CBB would be $Ag_xCs_{3-x}Bi_2Br_9$, where *x* denotes the concentration of Ag dopants, assuming that Ag_{ic}^{+} is the sole species of Ag present. We refrain from explicitly using this chemical formula, however, to prevent any misunderstanding that Ag directly substitutes onto the Cs sites.

For many defects, the preferred charge state may vary with respect to E_F, resulting in charge-state transition levels within the band gap. Such defects are often relevant for the absorption and emission of carriers. To analyze these species in more detail, we construct configuration coordinate diagrams,[4] for which we used tools from the Nonrad Python package.[5]

To relax defects, it is generally necessary to break the crystal symmetry. However, the ordered crystal structure of CBB, which is stable at room temperature, is dynamically unstable at 0 K; thus,

introducing defects and breaking the symmetry can result in unphysical lattice distortions even beyond the immediate vicinity of the defect. Because we are primarily concerned with properties of the Ag impurity at finite temperature, we therefore choose to restrict lattice distortions when calculating defects in the ordered structure. For comparison, we have also calculated defects in the ground state, disordered structure of CBB, which is 50 meV per formula unit lower in energy than the ordered structure at 0 K. The formation energies are generally consistent across the two structures, giving us confidence that our defect calculations in the ordered structure are accurate.

2.2. Chemical potentials

Chemical potentials are chosen to ensure the stability of CBB relative to various limiting phases. The chemical potentials are related to deviations $\Delta\mu_i$ from the energies of elemental ground states (bcc Cs, rhombohedral Bi, fcc Ag, and diatomic Br_2) as follows:

$$\mu_i = E_i + \Delta\mu_i.$$

To prevent these elemental phases from precipitating, we require that each $\Delta\mu_i \leq 0$. In addition, to ensure the thermodynamic stability of CBB, these deviations are related to its enthalpy of formation:

$$3\Delta\mu_{\mathrm{Cs}} + 2\Delta\mu_{\mathrm{Bi}} + 9\Delta\mu_{\mathrm{Br}} = \Delta H^f(\mathrm{Cs_3Bi_2Br_9}).$$

Other phases may form in certain chemical potential regimes, most notably the binary compounds CsBr and $BiBr_3$ (we have considered other compounds as well, but their formation will not be less likely). To prevent CsBr from precipitating, we require that:

$$\Delta\mu_{\mathrm{Cs}} + \Delta\mu_{\mathrm{Br}} \leq \Delta H^f(\mathrm{CsBr}),$$

and to prevent the formation of $BiBr_3$, we require that:

$$\Delta\mu_{\mathrm{Bi}} + 3\Delta\mu_{\mathrm{Br}} \leq \Delta H^f(\mathrm{BiBr_3}).$$

We can use the thermodynamic stability condition to reduce the overall stability region to two dimensions, which we arbitrarily chose to express in terms of $\Delta\mu_{\mathrm{Bi}}$ and $\Delta\mu_{\mathrm{Br}}$. After solving for $\Delta\mu_{\mathrm{Cs}}$ in terms of the other two chemical potentials, we rewrite the condition to prevent CsBr precipitation as:

$$\Delta\mu_{\mathrm{Bi}} + 3\Delta\mu_{\mathrm{Br}} \geq \frac{2}{3}\left(\Delta H^f(\mathrm{Cs_3Bi_2Br_9}) - \Delta H^f(\mathrm{CsBr})\right).$$

Our stability diagram is shown in Fig. S4. Various chemical potential regions of interest are labeled, and the precise chemical potentials are listed in Table S1. These can provide insights into defect formation under varieties of synthesis conditions. For the purposes of presentation, we will limit our discussion to intermediate chemical potentials in the main text.

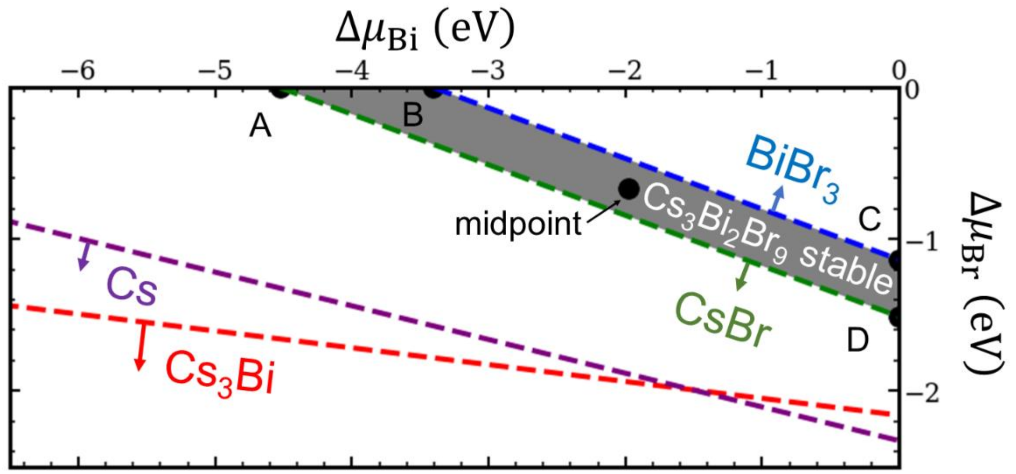

Figure S9. Thermodynamic stability of CBB. Chemical stability diagram for CBB, based on density functional theory calculations. The stability region is shaded gray. Selected chemical potential regions are labeled (A, B, C, D, and "midpoint"). Secondary phases become thermodynamically favored where indicated by colored dashed lines and arrows.

Table S1. Chemical potential conditions for CBB. Chemical potential values corresponding to the labeled points in Fig. S4. Ag chemical potentials correspond to the solubility limit of Ag-containing secondary phases.

**Chemical Potential Conditions**	$\Delta\mu_{Cs}$ (eV)	$\Delta\mu_{Bi}$ (eV)	$\Delta\mu_{Br}$ (eV)	$\Delta\mu_{Ag}$ (eV)
A: Equilibrium with Br_2 and CsBr	-3.97	-4.55	0.00	-1.30
B: Equilibrium with Br_2 and $BiBr_3$	-4.73	-3.40	0.00	-1.16
C: Equilibrium with Bi and $BiBr_3$	-3.60	0.00	-1.13	-0.02
D: Equilibrium with Bi and CsBr	-2.45	0.00	-1.52	0.00

midpoint	-3.69	-1.99	-0.66	-0.49

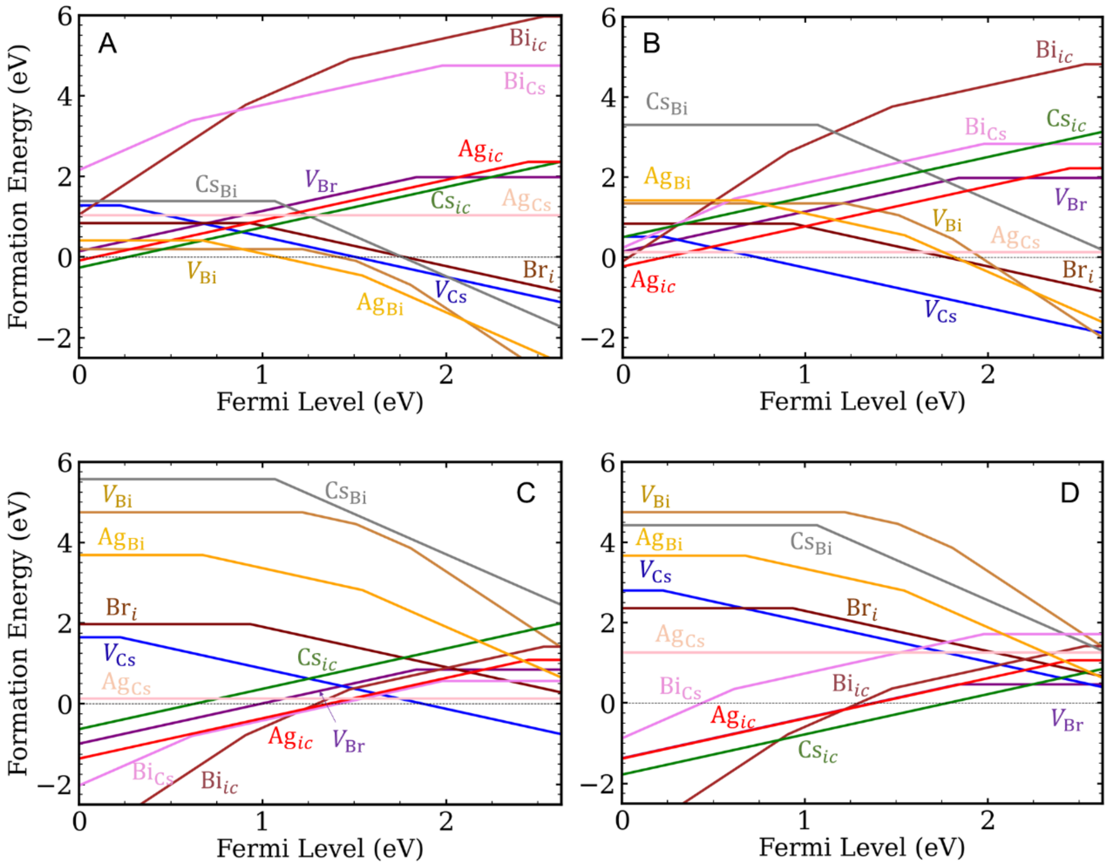

Figure S10. Formation energies of native point defects and silver impurities under a range of chemical potentials for disordered CBB. A, B, C, D and 'midpoint' correspond to chemical potential conditions labeled in Fig. S4 and Table S1.

Table S2. Concentrations of silver species in disordered CBB. Calculated Fermi level (E_F) position and corresponding concentrations of silver species (intercalated silver: Ag_{ic}^{+}; substitutional silver on a cesium site: $\mathrm{Ag}_{\mathrm{Cs}}^{0}$; and substitutional silver on a bismuth site: $\mathrm{Ag}_{\mathrm{Bi}}^{q}$) at different chemical potential conditions identified in Fig. S4 and Table S1.

**Chemical Potential Conditions**	$\boldsymbol{E_F}$ **(eV)**	$[\mathbf{Ag}_{ic}^{+}]$ **(at%)**	$[\mathbf{Ag}_{\mathbf{Cs}}^{\mathbf{0}}]$ **(at%)**	$[\mathbf{Ag}_{\mathbf{Bi}}^{q}]$ **(at%)**
A: Equilibrium with Br_2 and CsBr	0.67	1.40×10^{-8}	1.19×10^{-15}	2.56×10^{-5}
B: Equilibrium with Br_2 and $BiBr_3$	0.48	4.97×10^{-3}	2.01	2.63×10^{-22}
C: Equilibrium with Bi and $BiBr_3$	1.64	1.74×10^{-3}	2.01	1.97×10^{-42}
D: Equilibrium with Bi and CsBr	2.40	7.88×10^{-16}	2.34×10^{-19}	1.48×10^{-16}
midpoint	1.34	2.98×10^{-6}	7.24×10^{-7}	9.05×10^{-24}

References

1. Freysoldt, C.; Grabowski, B.; Hickel, T.; Neugebauer, J.; Kresse, G.; Janotti, A.; Van de Walle, C. G., First-Principles Calculations for Point Defects in Solids. *Reviews of Modern Physics* **2014**, *86*, 253-305.
2. Freysoldt, C.; Neugebauer, J.; Van de Walle, C. G., Fully Ab Initio Finite-Size Corrections for Charged-Defect Supercell Calculations. *Physical Review Letters* **2009**, *102*, 016402.
3. Freysoldt, C.; Neugebauer, J.; Van de Walle, C. G., Electrostatic Interactions between Charged Defects in Supercells. *physica status solidi (b)* **2011**, *248*, 1067-1076.

4. Alkauskas, A.; McCluskey, M. D.; Van de Walle, C. G., Tutorial: Defects in Semiconductors—Combining Experiment and Theory. *Journal of Applied Physics* **2016**, *119*, 181101.
5. Turiansky, M. E.; Alkauskas, A.; Engel, M.; Kresse, G.; Wickramaratne, D.; Shen, J.-X.; Dreyer, C. E.; Van de Walle, C. G., Nonrad: Computing Nonradiative Capture Coefficients from First Principles. *Computer Physics Communications* **2021**, *267*, 108056.
